# The APETALA2-Like Transcription Factor SUPERNUMERARY BRACT Controls Rice Seed Shattering and Seed Size

**DOI:** 10.1105/tpc.18.00304

**Published:** 2019-01-09

**Authors:** Liyun Jiang, Xin Ma, Shuangshuang Zhao, Yanyan Tang, Fengxia Liu, Ping Gu, Yongcai Fu, Zuofeng Zhu, Hongwei Cai, Chuanqing Sun, Lubin Tan

**Affiliations:** a National Center for Evaluation of Agricultural Wild Plants (Rice), MOE Laboratory of Crop Heterosis and Utilization, Department of Plant Genetics and Breeding, China Agricultural University, Beijing 100193, China; b State Key Laboratory of Plant Physiology and Biochemistry, China Agricultural University, Beijing 100193, China

## Abstract

The elimination of seed shattering was a crucial event during crop domestication. Improving and fine-tuning the regulation of this process will further enhance grain yield by avoiding seed losses during crop production. In this work, we identified the loss-of-shattering mutant *suppression of shattering1* (*ssh1*) through a screen of mutagenized wild rice (*Oryza rufipogon*) introgression lines with naturally high shattering. Using the MutMap approach and transformation experiments, we isolated a genetic factor for seed shattering, *SSH1*, which is an allele of *SUPERNUMERARY BRACT* (*SNB*), a gene encoding a plant-specific APETALA2-like transcription factor. A C-to-A point mutation in the ninth intron of *SNB* altered the splicing of its messenger RNA, causing the reduced shattering of the *ssh1* mutant by altering the development of the abscission layer and vascular bundle at the junction between the seed and the pedicel. Our data suggest that *SNB* positively regulates the expression of two rice *REPLUMLESS* orthologs, *qSH1* and *SH5*. In addition, the *ssh1* mutant had larger seeds and a higher grain weight, resulting from its increased elongation of the glume longitudinal cells. The further identification of favorable *SNB* alleles will be valuable for improving rice seed shattering and grain yield using molecular breeding strategies.

## Abstract (precis)

The APETALA2-like transcription factor SUPERNUMERARY BRACT confers seed shattering by positively regulating *qSH1* and *SH5*, while altering seed size by modulating longitudinal cell elongation in rice glumes.

## INTRODUCTION

Seed shattering is determined by the development of the abscission zone (AZ), which consists of several layers of isodiametrically flattened and thin-walled cells at the junction of the seed and the mother tissue (Jin, 1986; McKim et al., 2008). In the past, several important genes regulating the development of the AZ and the corresponding dispersal of plant organs have been identified in various species, shedding light on the regulatory mechanisms underlying these processes (Dong and Wang, 2015; Pourkheirandish et al., 2015; Ballester and Ferrándiz, 2017). The natural shattering of mature seeds has great ecological importance for offspring dispersal, whereas in domesticated crops, mutations that reduce shattering and cause the seeds to be retained longer on the parent plant were selected and fixed to facilitate efficient harvesting and avoid yield loss. The reduction of seed shattering was one of most important advances in the history of crop domestication (Fuller, 2007; Onishi et al., 2007).

Previous studies have identified several genetic factors controlling the key transition from shattering to nonshattering during crop domestication. Rice (*Oryza sativa*) was one of the first crops to be domesticated. In this species, mutations in two genes, *SH4* (the quantitative trait locus *seed shattering on chromosome4*)/*SHA1* (*Shattering 1*, an allele of *SH4*) and *qSH1* (the quantitative trait locus *seed shattering on chromosome1*), were mainly responsible for the transition away from seed shattering during domestication (Konishi et al., 2006; Li et al., 2006; Lin et al., 2007). A single amino acid change in the MYB3 DNA binding domain of SH4 resulted in decreased seed shattering due to the incomplete formation of the AZ in cultivated rice (Li et al., 2006). A single nucleotide polymorphism (SNP) in the 5′ regulatory region of *qSH1*, a rice ortholog of the BEL1-type homeobox gene *REPLUMLESS* (*RPL*) in Arabidopsis (*Arabidopsis thaliana*), caused the absence of the abscission layer in temperate *japonica* varieties (Konishi et al., 2006). The genetic control of seed shattering domestication has also been revealed for other species, including wheat (*Triticum aestivum*), barley (*Hordeum vulgare*), sorghum (*Sorghum bicolor*), and soybean (*Glycine max*; Faris and Gill, 2002; Faris et al., 2003; Simons et al., 2006; Zhang et al., 2011, 2013; Lin et al., 2012; Dong et al., 2014; Funatsuki et al., 2014; Katkout et al., 2015; Pourkheirandish et al., 2015; Debernardi et al., 2017). These findings provide important insights into the molecular mechanisms and evolutionary trajectories underlying seed shattering domestication.

Notably, some genes affecting seed shattering have a pleiotropic effect on spikelet development; for example, in African cultivated rice (*Oryza glaberrima*), the selection of an independent *sh4* mutant resulted in the convergent evolution of the nonshattering trait observed in Asian cultivated rice (*O. sativa*); however, the *sh4* mutant allele in African rice resulted in smaller seeds than its progenitor *Oryza barthii*, owing to a reduced elongation of the glume longitudinal cells (Wu et al., 2017). The mutation of *SHATTERING ABORTION1* (*SHAT1*) caused both the loss of shattering and spikelet developmental defects in rice (Zhou et al., 2012). In addition, the mutation of a wheat domestication gene, *Q*, encoding an APETALA2 (AP2) transcription factor, affects a broad range of phenotypic characters, including subcompact inflorescences, glume shape, and free-threshing grains (Faris and Gill, 2002; Faris et al., 2003; Simons et al., 2006; Debernardi et al., 2017). The further identification of other nonshattering mutants would therefore facilitate the elucidation of the molecular basis underlying seed shattering while also providing a novel favorable genetic resource for high-yielding crop breeding programs.

**Figure:**
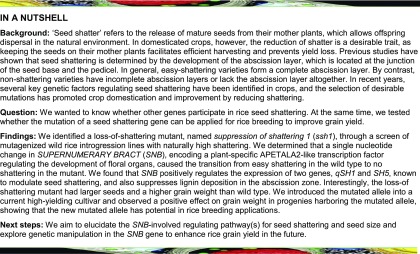


In the present study, we identified a genetic suppressor of seed shattering, *SUPPRESSION OF SHATTERING1* (*SSH1*), in a screen of mutagenized wild rice (*Oryza rufipogon*) introgression lines with naturally high levels of shattering, using a combination of MutMap analysis and transformation experiments. *SSH1*, which is an allele of *SUPERNUMERARY BRACT* (*SNB*) encoding a transcription factor with two plant-specific AP2 domains, controls the development of the AZ and regulates seed shattering by promoting the expression of two rice *RPL* orthologs, *qSH1* and *SH5*. In addition, the *ssh1* mutant identified in this study has larger seeds and a higher grain yield, suggesting that this gene may be a useful target in rice breeding applications.

## RESULTS

### Identification of a Loss-of-Shattering Mutant from Mutagenized Wild Rice Introgression Lines with Naturally High Shattering

**Figure 1.**
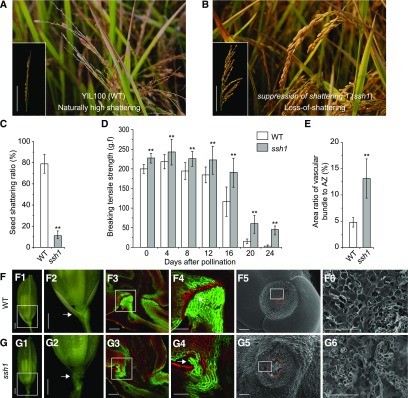
Comparison of Seed Shattering and Floral AZ Morphology in the Wild Type and the *ssh1* Mutant. **(A)** and **(B)** Phenotypes of mature wild-type (YIL100) and *ssh1* rice in the field. Panicles of the wild type and the *ssh1* mutant are respectively illustrated in the lower left corners. Bars = 10 cm. **(C)** Comparison of the seed shattering ratio of the mature wild type and the *ssh1* mutant. Values are means ± sd (*n* = 10 main panicles). **(D)** Comparison of the BTS in the wild type and the *ssh1* mutant on the day after pollination and every 4 d thereafter during seed development. The g.f is the gravitational unit of force. Values are means ± sd (*n* = 50 grains). **(E)** Comparison of the area ratio of the vascular bundle to AZ in the wild type and the *ssh1* mutant. Values are means ± sd (*n* = 10 grains). In **(C)** to **(E)**, two-tailed Student’s *t* tests were used to compare the wild type and the *ssh1* mutant (**, P < 0.01; Supplemental File 2). **(F)** and **(G)** Characterization of floral AZ morphology in the wild type **(F)** and the *ssh1* mutant **(G)**. **(F1)** and **(G1)** show the spikelets. The white boxes indicate the junction between the seed and the pedicel and are enlarged in **(F2)** and **(G2)**, where the arrows indicate the position of the AZ. **(F3)** and **(G3)** show the fluorescence images of longitudinal sections across the flower and pedicel junction. **(F4)** and **(G4)** are enlargements of the white boxes in **(F3)** and **(G3)**, respectively. Arrows point to the AZ in the wild type or the corresponding region in the *ssh1* mutant. **(F5)** and **(G5)** show SEM photographs of the seed bases where the pedicels attach. The red circles indicate vascular bundles. **(F6)** and **(G6)** are magnifications of the white boxes in **(F5)** and **(G5)**, respectively. The surface of the seed base in the wild type is smooth, whereas the surfaces in the *ssh1* mutant are broken and rough. Bars = 1 mm in panels **(1)** and **(2)**, 100 μm in panels **(3)** and **(5)**, and 50 μm in panels **(4)** and **(6)**.

A wild rice introgression line, YIL100, referred to as the wild type below, was derived from the cross between the donor, *O. rufipogon* (accession YJCWR), and the recipient, an *indica* variety known as Teqing. The YIL100 introgression line carried *SH4*/*SHA1* from the donor and the *qSH1* locus from the recipient, resulting in a natural seed shattering phenotype (Figure 1A; Supplemental Figure 1). To study the regulatory mechanism underlying seed shattering in rice, we mutagenized the YIL100 line using EMS and screened the resulting M2 mutant generation, identifying the loss-of-shattering mutant *ssh1* (Figure 1B). Phenotypic observation showed that the *ssh1* mutant had less seed shattering at the harvest stage and a stronger pedicel breaking tensile strength (BTS) than the wild type (Figures 1C and 1D). Further observation of longitudinal sections of spikelets at the anthesis stage using confocal microscopy showed that the wild type had a complete AZ and a normal vascular bundle at the junction between the seed and pedicel, while the *ssh1* mutant displayed a deficiency in AZ development and vascular bundle overgrowth (Figures 1F and 1G). The seed bases were observed using a scanning electron microscope (SEM), further revealing that the *ssh1* mutation affected the development of both the abscission layer and the vascular bundle (Figures 1E to 1G). These results suggest that both the incomplete abscission layer and the larger vascular bundle in the *ssh1* mutant might provide stronger support to the seed, decreasing shattering after seed ripening.

### Cloning and Characterization of *SSH1*

**Figure 2.**
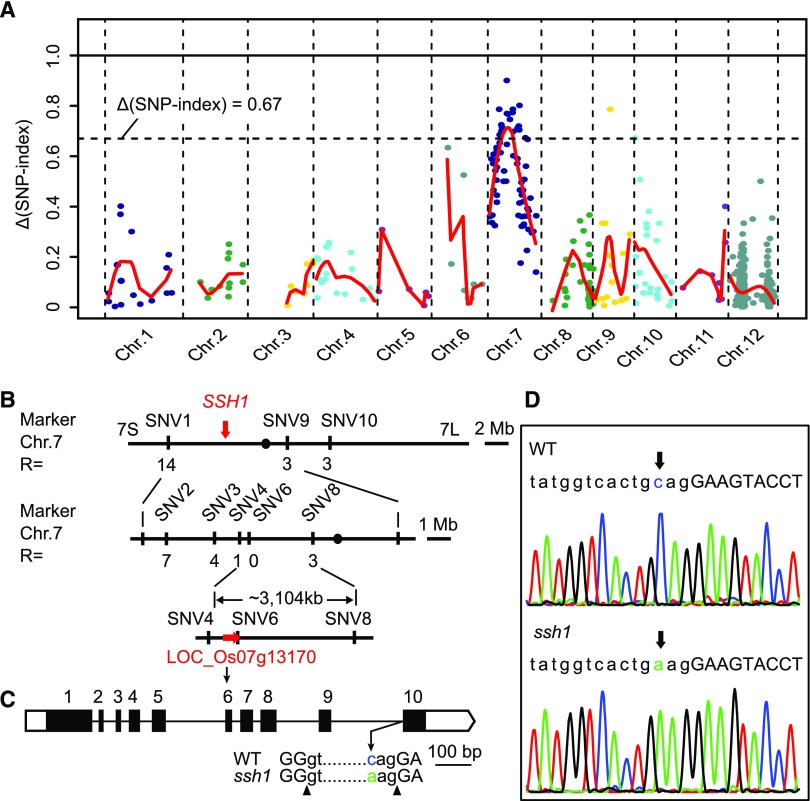
Mapping of *SSH1*. **(A)** Δ(SNP-index) plot of the whole genome generated using MutMap. The red curves represent Δ(SNP-index) plot regression lines. **(B)** The mapping location of the *SSH1* gene was narrowed down to an ∼3104-kb interval between SNP markers SNV4 and SNV8 using 72 recessive F2 individuals. It was found to cosegregate with the SNP SNV6, which is located in the ninth intron of LOC_Os07g13170. The vertical red arrow indicates the location of *SSH1*. The horizontal red arrow represents the gene LOC_Os07g13170. **(C)** The exon-intron structure of LOC_Os07g13170 and the location of the SNP SNV6. Black boxes represent exons, white boxes represent UTRs, and thin black lines indicate introns. Uppercase letters and lowercase letters below the gene structure represent exons and introns, respectively. Blue and green lowercase letters represent the C in the wild type and the A in the *ssh1* mutant, respectively. Black triangles indicate the 5′ and 3′ splicing sites of the ninth intron of LOC_Os07g13170. **(D)** The C-to-A point mutation (SNV6) in LOC_Os07g13170, detected using Sanger sequencing. Lowercase letters and uppercase letters above the DNA sequencing peak chromatograms represent the 9th intron and the 10th exon of LOC_Os07g13170, respectively.

To evaluate the genetic characteristics of the phenotypes affected in the *ssh1* mutant, we developed an F2 population derived from a cross between the wild type and the *ssh1* mutant. Phenotypic observation of the F2 population (205 wild-type plants and 72 mutant plants; χ^2^ = 0.10 < χ^2^_0.05,1_ = 3.84) revealed that nonshattering in *ssh1* was controlled by a single recessive gene. We further bulked DNA from 20 recessive homozygous nonshattering plants and 20 dominant plants with natural shattering and then sequenced the pooled DNA on an Illumina HiSeq2500 platform. Using the MutMap approach (Abe et al., 2012; Takagi et al., 2013; Lu et al., 2014), a single peak was detected on chromosome 7, in which the Δ(SNP-index) was more than 0.67, indicating that the SNPs are associated with *SSH1* (Figure 2A; Supplemental Table 1). Using a total of 72 recessive homozygous plants from the same F2 population, *SSH1* was further mapped to an ∼3104-kb interval between SNP markers SNV4 and SNV8 and was found to cosegregate with SNV6 (Figure 2B).

Integrating data from whole-genome sequencing and SNP validation using Sanger sequencing, we identified three SNPs between the wild type and *ssh1* mutant within the 3104-kb *SSH1* fine-mapped region (Supplemental Table 1). We further compared the expression levels of four genes associated with these three SNPs in the wild type and *ssh1* mutant using RNA-seq. The expression of LOC_Os07g13170 was significantly downregulated in *ssh1* compared with the wild type (fold change ≥ 2, false discovery rate [FDR] < 0.001), while the expression levels of the three genes associated with the other two SNPs were not significantly different between the *ssh1* mutant and the wild type (Supplemental Table 1). Based on the annotation of the rice reference genome (http://rice.plantbiology.msu.edu), we found that the SNP SNV6, a single nucleotide transversion from cytosine (C) to adenine (A) in the *ssh1* mutant, was located in the ninth intron (+3473 bp) of LOC_Os07g13170 (Figures 2C and 2D). Therefore, we speculated that the SNP SNV6 might alter the expression profile of LOC_Os07g13170. Additionally, LOC_Os07g13170 is the rice heterochronic gene *SNB*, which encodes an AP2 domain-containing protein regulating the transition from the spikelet meristem to the floral meristem (Lee et al., 2007; Zhu et al., 2009; Lee and An, 2012). Taken together, these results suggest that LOC_Os07g13170 is a strong candidate gene for *SSH1*.

**Figure 3.**
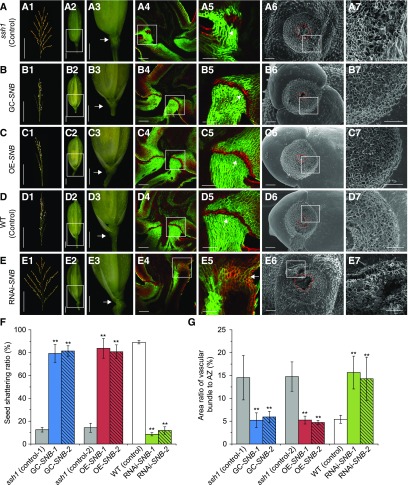
Characterization of Seed Shattering in the GC-*SNB*, OE-*SNB*, and RNAi-*SNB* Transgenic Plants. **(A)** to **(E)** The morphological analyses of the *ssh1* mutant (control; **[A]**), the GC-*SNB* transgenic plants **(B)**, the OE-*SNB* transgenic plants **(C)**, the wild type (control; **[D]**), and the RNAi-*SNB* transgenic plants **(E)**. **(A1)**, **(B1)**, **(C1)**, **(D1)**, and **(E1)** show the seed shattering phenotypes on the main panicle. **(A2)**, **(B2)**, **(C2)**, **(D2)**, and **(E2)** show the spikelets. The white boxes indicating the junction between the seed and the pedicel are magnified in **(A3)**, **(B3)**, **(C3)**, **(D3)**, and **(E3)**, respectively, in which arrows indicate the position of the AZ. **(A4)**, **(B4)**, **(C4)**, **(D4)**, and **(E4)** show fluorescence images of longitudinal sections across the flower and pedicel junction. The white boxes are magnified in **(A5)**, **(B5)**, **(C5)**, **(D5)**, and **(E5)**, respectively, and the arrows point to the AZ or corresponding regions. **(A6)**, **(B6)**, **(C6)**, **(D6)**, and **(E6)** are SEM photographs of the seed base. The red circles indicate the vascular bundles. **(A7)**, **(B7)**, **(C7)**, **(D7)**, and **(E7)** are magnifications of the white boxes in **(A6)**, **(B6)**, **(C6)**, **(D6)**, and **(E6)**, respectively. Bars = 50 μm in panels **(5)** and **(7)**, 100 μm in panels **(4)** and **(6)**, 1 mm in panels **(2)** and **(3)**, and 10 cm in panel **(1)**. **(F)** Comparison of seed shattering ratios. Values are means ± sd (*n* = 10 main panicles). **(G)** Comparison of the area ratios of vascular bundle to the AZ of the seed bases. Values are means ± sd (*n* = 10 grains). In **(F)** and **(G)**, two-tailed Student’s *t* tests were used to compare the transgenic lines and the corresponding controls (**, P < 0.01; Supplemental File 2).

To test whether the phenotype of the *ssh1* mutant was caused by this SNP in the *SNB* gene, we complemented the mutant with a 7206-bp wild-type genomic fragment containing the entire coding region of *SNB* and the 2309-bp 5′-flanking and 1209-bp 3′-flanking regions. A total of 11 independent positive transgenic complementation lines (GC-*SNB*) were generated, all of which had natural shattering and normal development of both the abscission layer and the vascular bundle at the junction between the seed and the pedicel, indicating complementation of the *ssh1* phenotype (Figures 3A, 3B, 3F, and 3G). We also developed both overexpression and RNA interference (RNAi) constructs based on the *SNB* (LOC_Os07g13170) complementary DNA (cDNA) sequence from the wild type and introduced these into the *ssh1* mutant and the wild-type plants, respectively. We found that all 17 independent overexpression transgenic plants (OE-*SNB*) showed an integral abscission layer, smaller vascular bundles at the seed base, and increased shattering compared with the *ssh1* mutant (Figures 3C, 3F, and 3G). By contrast, all eight independent RNAi transgenic plants (RNAi-*SNB*) with significantly downregulated *SNB* transcripts (Supplemental Figure 2) showed a deficiency in AZ development, larger vascular bundles, and decreased shattering compared with the wild type, displaying similar phenotypes to the *ssh1* mutants (Figures 3D to 3G). The results of these transformation experiments demonstrate that the C-to-A point mutation in the intron of *SNB* resulted in defective abscission layer development and vascular bundle overgrowth, leading to a decrease of seed shattering in rice.

### *SNB*/*SSH1* Encodes a Transcription Factor with Two AP2 Domains

**Figure 4.**
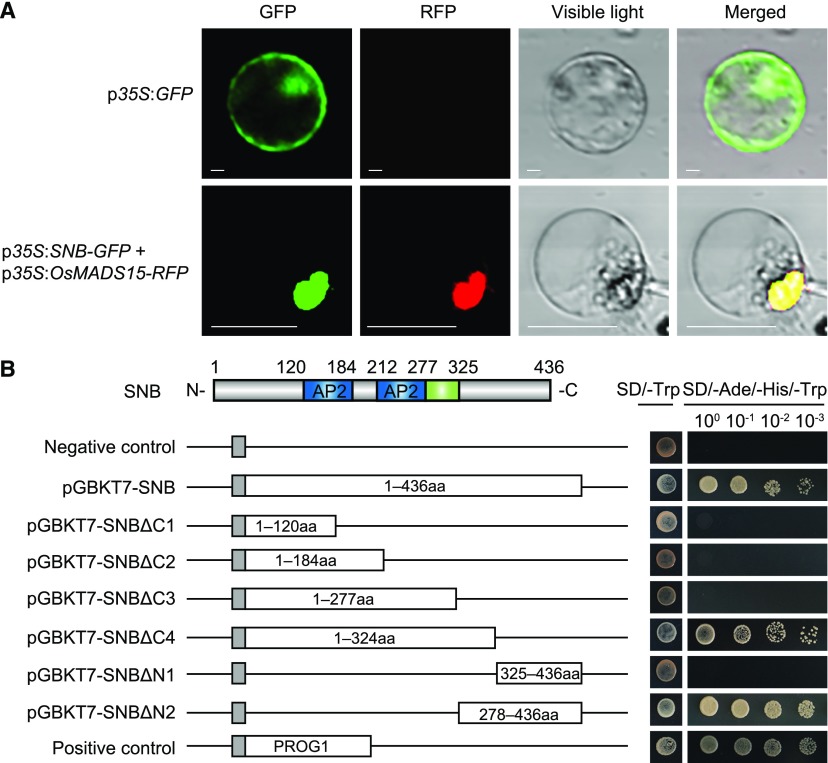
Subcellular Localization and Transcription Activity of SNB. **(A)** Subcellular localization of the SNB-GFP fusion protein in a rice protoplast. The OsMADS15-RFP fusion protein was used as a nuclear localization marker, and the GFP protein alone was used as the control. Bars = 20 μm. **(B)** Transcription activity assay of full-length or truncated SNB in yeast. pGBKT7-SNB, pGBKT7-SNBΔC (1–4), and pGBKT7-SNBΔN (1 and 2) had the GAL4 BD (gray) in the pGBKT7 vector fused with sequences encoding full-length, N-terminal, or C-terminal SNB, respectively. pGBKT7 was used as the negative control, and the transcription factor PROSTRATE GROWTH1 was fused with the GAL4 BD as the positive control. The numbers in the boxes indicate the SNB amino acid residues used for construction.

The full-length cDNA of *SNB*/*SSH1* is 1909 bp and is divided into 10 exons interspersed with 9 introns (Supplemental Figure 3). The open reading frame of *SNB* is 1311 bp and encodes a protein of 436 amino acid residues, forming two plant-specific AP2 domains between residues 121 and 184 and residues 213 and 277, respectively (Supplemental Figure 4). Transient expression experiments indicated that the SNB-green fluorescent protein (GFP) fusion protein was specifically localized to the nucleus in rice protoplasts (Figure 4A). A transcriptional activation assay showed that the SNB and DNA binding domain (BD) fusion protein in yeast activated the expression of the reporter genes, implying that SNB has strong transcriptional activity and is a transcription factor. A truncation analysis revealed that residues 278 to 324 of SNB are required for its transcriptional activity (Figure 4B). A phylogenetic analysis showed that SNB is a rice ortholog of SID1 (SISTER OF INDETERMINATE SPIKELET1) in maize (*Zea mays*; Chuck et al., 2008) and has high amino acid similarity to rice OsIDS1 (Lee et al., 2007), maize IDS1 (INDETERMINATE SPIKELET1; Chuck et al., 1998), and wheat Q (Supplemental Figure 4; Supplemental File 1; Faris et al., 2003), indicating that the AP2 transcription factors have conserved functions in regulating the development of the AZ and inflorescences in cereal crops.

### Posttranscriptional Regulation of *SNB*

The mutant gene *ssh1* identified in this study is a novel allele of *SNB*. Compared with the knockout *snb* mutant (Lee et al., 2007), *ssh1* had a weaker pleiotropic phenotype for both inflorescence and spikelet development, including forming fewer rudimentary glumes (2.9% in *ssh1*, 31.3% in *snb*), aberrant lemma/palea-like structures (8.7% in *ssh1*, 35.6% in *snb*), and ectopic lodicules (2.3% in *ssh1*, 14.3% in *snb*; Table 1; Supplemental Figure 5). We therefore speculated that the C-to-A point mutation (SNV6) in the ninth intron of *SNB* in the *ssh1* mutant might alter its transcription level or messenger RNA (mRNA) splicing rather than cause a complete loss of function, which is consistent with the weaker phenotype of *ssh1* than the loss-of-function *snb* mutant.

**Table 1.** Comparison of Floral Organ Numbers in the Wild Type and the *ssh1* Mutant.

No. of Organs	Glume		Palea and Lemma		Lodicule		Stamen		Carpel		Stigma	
	Wild Type	*ssh1*	Wild Type	*ssh1*	Wild Type	*ssh1*	Wild Type	*ssh1*	Wild Type	*ssh1*	Wild Type	*ssh1*
0	–	–	–	–	–	12	–	13	–	13	–	13
1	–	–	–	6	–	–	–	1	516	495	–	–
2	–	–	516	471	516	492	–	5	–	8	516	494
3	–	–	–	30	–	6	–	3	–	–	–	1
4	516	502	–	8	–	3	–	6	–	–	–	7
5	–	11	–	–	–	1	–	6	–	–	–	–
6	–	1	–	1	–	–	516	480	–	–	–	1
7	–	1	–	–	–	1	–	–	–	–	–	–
8	–	1	–	–	–	1	–	2	–	–	–	–

A total of 516 spikelets each were investigated in the wild type and the *ssh1* mutant. The dashes indicate no spikelets showing the corresponding phenotype.

**Figure 5.**
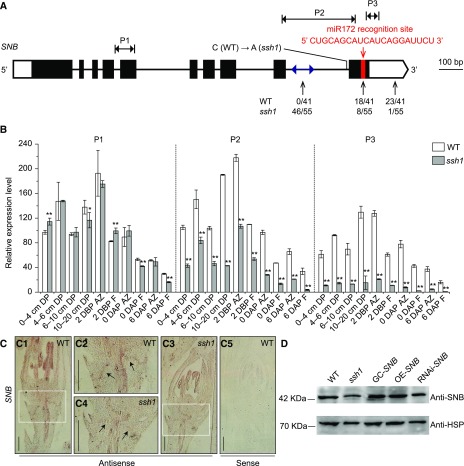
The Point Mutation SNV6 Alters the mRNA Splicing of *SNB*. **(A)** The posttranscriptional regulation of *SNB* caused by the SNP SNV6 and miR172. The 3′ end of *SNB*/*ssh1* was determined by cloning and sequencing the amplicons generated using RACE. The blue double-sided arrow in the 9th intron represents the randomly distributed region of the 3′ ends of *SNB* transcripts detected in the *ssh1* mutant. The red bar in the 10th exon indicates the miR172 target site. The black vertical arrows indicate the inferred 3′ ends of the *SNB*/*ssh1* mRNAs detected in the wild type and the *ssh1* mutant. The numbers below each arrow indicate the proportion of clones that possess these sites. The regions were targeted using RT-qPCR with three primer sets, P1, P2, and P3, indicated by the black double-sided arrows. **(B)** Comparison of *SNB* expression in various tissues of the wild type and the *ssh1* mutant using RT-qPCR with three primer sets, P1, P2, and P3 (*n* = 3 pooled tissues, five plants per pool). DP, developing panicles; F, florets; DBP, day before pollination; DAP, day after pollination. The rice housekeeping gene *UBIQUITIN* was used as an internal control to normalize gene expression data. Values are means ± sd. Two-tailed Student’s *t* tests were used to compare the wild type and the *ssh1* mutant (*, P < 0.05 and **, P < 0.01; Supplemental File 2). **(C)** mRNA in situ hybridization of *SNB* at spikelet developmental stage Sp8. **(C1)** and **(C3)** show that stronger *SNB* expression is maintained in the spikelet of the wild type than the *ssh1* mutant. **(C2)** and **(C4)** are magnifications of the white boxes in **(C1)** and **(C3)**, respectively, in which arrows indicate the abscission layer. **(C5)** shows a negative control using the sense probe. Bars = 200 μm. **(D)** Immunoblot analysis of SNB in the wild type, the *ssh1* mutant, GC-*SNB*, OE-*SNB*, and RNAi-*SNB*. The rice housekeeping protein HSP served as the loading control.

Like other *AP2*-like genes, previous studies revealed that the transcript levels of *SNB* are negatively regulated by a microRNA; microRNA172 (miR172) recognizes the *SNB* mRNA sequence at 1196 to 1216 bp (Lee et al., 2007; Zhu et al., 2009; Lee and An, 2012; Wang et al., 2015). To distinguish the effects of the SNP SNV6 and miR172 on *SNB* expression, we performed an reverse transcription quantitative PCR (RT-qPCR analysis to detect the expression levels of *SNB* using three primer sets (P1, P2, and P3; locations shown in Figure 5A). RT-qPCR analysis using primer set P1 (spanning the fourth intron) showed that the expression of *SNB* was similar between the *ssh1* mutant and the wild-type plants (Figure 5B). This indicates that the SNP in the *ssh1* mutant did not affect the transcription of this gene. In addition, among the organs investigated, *SNB* expression was highest in the AZ (the junctions between the seed and the pedicel) at 2 d before pollination, which is consistent with its function in regulating AZ development.

RT-qPCR analysis using primer set P2 (spanning the 9th intron) indicated significantly lower levels of *SNB* transcripts in the *ssh1* mutant than in the wild type, indicating that the SNP SNV6 might alter mRNA splicing (Figure 5B). Additionally, the expression levels of *SNB* detected using primer set P3 (across the target site of miR172) were lower than those detected using P2 in both the *ssh1* mutant and the wild type, implying that miR172 negatively regulates the expression of *SNB* by facilitating mRNA cleavage (Figure 5B). A further rapid amplification of cDNA ends (RACE) experiment was performed to investigate the 3′ ends of the *SNB* transcripts in both the *ssh1* mutant and the wild type. Sequencing the clones of the RACE amplicons revealed that, in the wild type, 56.1% (23/41) and 43.9% (18/41) of the 3′ ends of the *SNB* transcripts were located in the 3′-untranslated region (UTR) and the miR172 target site, respectively (Figure 5A; Supplemental Figure 6), while in the *ssh1* mutant, only 1.8% (1/55) and 14.5% (8/55) were located at the 3′-UTR and the miR172 target site, respectively, with the remaining (83.6%) distributed randomly throughout the 9th intron. These results indicate that the SNP SNV6, which is close to the 3′ acceptor site between the 9th intron and the 10th exon, caused aberrant splicing of the 9th intron in the *ssh1* mutant (Figure 5A; Supplemental Figure 6).

An in situ hybridization analysis using an antisense probe designed to target the 3′-subterminal region of *SNB* was used to compare the transcript levels of *SNB* in the *ssh1* mutant and the wild-type plants. We found that *SNB* was expressed in both the anthers and the AZ in the *ssh1* mutant, but to a lesser extent than in the wild type (Figure 5C). Additionally, immunoblot analysis showed that the *ssh1* mutant produced less SNB protein than the wild type, consistent with the decrease in the abundance of full-length *SNB* transcript (Figure 5D). Taken together, the SNP SNV6 reduces the number of entire *SNB* transcripts in *ssh1* by altering its mRNA splicing, leading to a decrease in seed shattering.

### *SNB* Positively Regulates the Expression of Two Rice *RPL* Orthologs, *qSH1* and *SH5*

**Figure 6.**
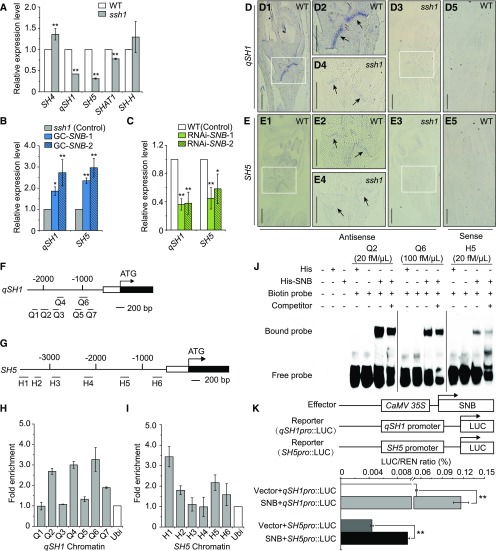
*SNB* Positively Regulates the Expression of *qSH1* and *SH5*. **(A)** Comparison of the expression levels of five rice seed shattering genes in 0- to 4-cm young panicles of the wild type and the *ssh1* mutant. The rice housekeeping gene *UBIQUITIN* was used as an internal control to normalize gene expression data. Values are means ± sd (*n* = 3 pooled tissues, five plants per pool). Two-tailed Student’s *t* tests were used to compare the wild type and the *ssh1* mutant (**, P < 0.01). **(B)** Comparison of expression levels of *qSH1* and *SH5* in the young panicles (0–4 cm) of the GC-*SNB* lines and the *ssh1* mutant (control). **(C)** Comparison of the expression levels of *qSH1* and *SH5* in the young panicles (0–4 cm) of the RNAi-*SNB* plants and the wild type (control). In **(B)** and **(C)**, the rice housekeeping gene *UBIQUITIN* was used as an internal control to normalize gene expression data. Values are means ± sd (*n* = 3 pooled tissues, five plants per pool). Two-tailed Student’s *t* tests were used to compare the transgenic lines and the corresponding controls (*, P < 0.05 and **, P < 0.01; Supplemental File 2). **(D)** and **(E)** Stronger mRNA hybridization signals of *qSH1* **(D)** and *SH5* **(E)** are detected in the AZ of the wild type than the *ssh1* mutant. **(D2)**, **(D4)**, **(E2)**, and **(E4)** are magnifications of the white boxes in **(D1)**, **(D3)**, **(E1)**, and **(E3)**, respectively, and the arrows indicate the abscission layer. **(D5)** and **(E5)** show the hybridization signal using the sense probes as a negative control. Bars = 200 μm. **(F)** to **(I)** ChIP-qPCR assays of *qSH1* and *SH5* using ChIP-DNA complexes isolated from 0- to 4-cm young panicles of the SNB-GFP transgenic plants. **(F)** and **(G)** show the genomic structures of *qSH1* and *SH5*, respectively. The numbers (Q1 to Q7 and H1 to H6) indicate the tested regions. **(H)** and **(I)** show the enrichment of SNB on the *qSH1* and *SH5* chromatin, indicated as the fold change in the immunoprecipitation sample over the control containing no antibodies. Values are means ± sd (*n* = 3 pooled tissues, 10 plants per pool). **(J)** EMSA revealed that the His-SNB recombinant protein was able to bind to both the Q2 and Q6 fragments of the *qSH1* promoter and the H5 fragment of the *SH5* promoter. **(K)** Dual luciferase reporter assays in rice protoplasts showed that the SNB protein promoted the expression of the *LUC* gene through binding to the *qSH1* and *SH5* promoters. Vector represents the pGreenII 62-SK empty vector, and REN represents the *Renilla luciferase* gene. Values are means ± sd (*n* = 3 biological replicates). Difference significance analysis was conducted with two-tailed Student’s *t* tests (**, P < 0.01; Supplemental File 2).

To elucidate the regulatory pathway involving *SNB*, we compared the expression of the five known rice genes related to seed shattering in the *ssh1* mutant (Supplemental Figure 7). Compared with the wild type, the two rice orthologs of *RPL*, *qSH1* and *SH5*, were dramatically downregulated in the young panicles (0–4 cm, the stage of abscission layer formation) of the *ssh1* mutant (Figure 6A). We further investigated the expression levels of *qSH1* and *SH5* in the GC-*SNB* and the RNAi-*SNB* lines. Consistent with the changes in *qSH1* and *SH5* expression between *ssh1* and the wild type, both *qSH1* and *SH5* were significantly upregulated and downregulated in the GC-*SNB* and RNAi-*SNB* lines, respectively, compared with their controls (Figures 6B and 6C). In addition, an mRNA in situ hybridization demonstrated that the transcripts of both *qSH1* and *SH5* were reduced in the AZ of the *ssh1* mutant compared with the wild type (Figures 6D and 6E). These results indicated that *SNB* positively regulates the expression of *qSH1* and *SH5*.

To examine whether *SNB* affects the development of the abscission layer by directly regulating *qSH1* and *SH5* in vivo, we first generated a construct containing an *SNB-GFP* fusion gene under the control of the *Cauliflower mosaic virus* (*CaMV*) *35S* promoter and introduced it into the *ssh1* mutant. The transgenic plants constitutively expressing the *SNB*-*GFP* fusion gene (termed SNB-GFP) had wild-type levels of seed shattering, implying that the SNB-GFP fusion protein had a similar function to SNB (Supplemental Figure 8). Furthermore, we performed a chromatin immunoprecipitation qPCR (ChIP-qPCR) assay using 0- to 4-cm young panicles from the SNB-GFP transgenic plants. Our data showed that the DNA fragments were more than 3-fold enriched at the promoter (Q2, Q4, and Q6) of the *qSH1* gene and the promoter (H1) of the *SH5* gene (Figures 6F to 6I). We subsequently performed an electrophoretic mobility shift assay (EMSA) using the His-SNB recombinant protein to determine whether the promoters of *qSH1* and *SH5* were bound by SNB in vitro. The probes were synthesized based on the nucleotide sequences of the DNA fragments (Q1 through Q7 and H1 through H6) and labeled with biotin at the 3ʹ end, while the unlabeled probes were used as competitors. The EMSA revealed that the His-SNB recombinant protein was able to bind to both the Q2 and Q6 fragments of the *qSH1* promoter and the H5 fragment of the *SH5* promoter (Figure 6J; Supplemental Figure 9). Additionally, dual luciferase reporter assays in rice protoplasts showed that *SNB* can promote the expression of the *LUC* gene through binding to both the *qSH1* and *SH5* promoters (Figure 6K). These results suggest that *SNB* controls the development of the AZ by directly regulating the expression of two rice *RPL* orthologs, *qSH1* and *SH5*.

**Figure 7.**
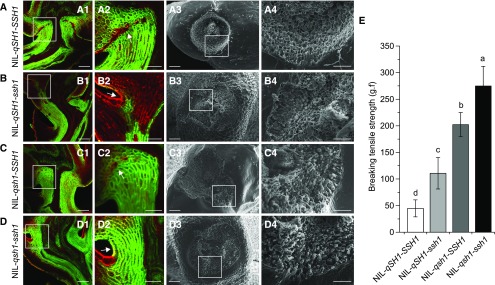
Characterization of Floral AZ Morphology in the Four NILs of *qSH1* and *SNB*. **(A)** to **(D)** Morphological analyses of NIL*-qSH1-SSH1* **(A)**, NIL*-qSH1-ssh1* **(B)**, NIL*-qsh1-SSH1* **(C)**, and NIL*-qsh1-ssh1* **(D)**. **(A1)**, **(B1)**, **(C1)**, and **(D1)** show fluorescence images of longitudinal sections across the flower and pedicel junction. **(A2)**, **(B2)**, **(C2)**, and **(D2)** are magnifications of the white boxes in **(A1)**, **(B1)**, **(C1)**, and **(D1)**, respectively, and the arrows point to the AZ or the corresponding region. **(A3)**, **(B3)**, **(C3)**, and **(D3)** show SEM images of the seed base. The white boxes in these images are magnified in **(A4)**, **(B4)**, **(C4)**, and **(D4)**, respectively. Bars = 50 μm in panels **(2)** and **(4)** and 100 μm in panels **(1)** and **(3)**. **(E)** Comparison of BTS in the four NILs at 35 d after pollination. Values are means ± sd (*n* = 50 grains). The g.f is the gravitational unit of force. Different letters denote significant differences (P < 0.01) determined using Tukey’s honestly significant difference analysis (Supplemental File 2).

To investigate the AZ developmental effects of the regulatory pathway involving *SNB*/*SSH1* and *qSH1*, we further developed four near-isogenic lines (NILs) in the genetic background of the *indica* variety Teqing with the inactive *SH4* gene: NIL-*qSH1*-*SSH1*, NIL-*qSH1*-*ssh1*, NIL-*qsh1*-*SSH1*, and NIL-*qsh1*-*ssh1* (Supplemental Figure 10). Longitudinal sections of the spikelets at anthesis were observed using confocal microscopy, which revealed that, in NIL-*qSH1*-*SSH1*, the AZ cells were formed in the basal area near the sterile lemmas, but the abscission layer was discontinuous (Figure 7A). In NIL-*qSH1*-*ssh1*, the formation of the AZ cells was repressed on one side, indicating defective AZ development (Figure 7B). The AZ cells of NIL-*qsh1*-*SSH1* were visible but their development was incomplete (Figure 7C). In NIL-*qsh1*-*ssh1*, the abscission layer was completely absent, the surfaces of the seed base were broken and rough, and the pedicel BTS was the strongest among the four NILs (Figures 7D and 7E), indicating that the mutation of *SNB* in NIL-*qsh1*-*ssh1* might cause a lower level of *qSH1* expression than was present in NIL-*qsh1*-*SSH1*, completely suppressing the development of the AZ.

### *SNB* Affects Lignin Deposition in the AZ

**Figure 8.**
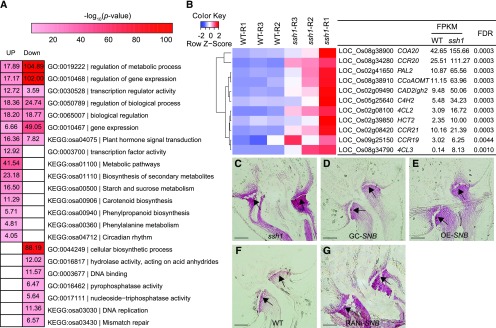
*SNB* Negatively Regulates Lignin Biosynthesis and Metabolism. **(A)** GO and KEGG analysis of 1403 upregulated and 999 downregulated genes in the young panicles (0–4 cm) of the wild type and the *ssh1* mutant. **(B)** Hierarchical clustering of 11 DEGs involved in lignin biosynthesis. The average fragments per kilobase of exon per million mapped reads (FPKM) value of all three biological replicates is shown. **(C)** to **(G)** Comparison of lignin deposition in the pedicel junction at spikelet developmental stage Sp8, revealed using phloroglucinol staining. The arrows indicate the AZ. Bars = 100 μm.

To analyze the molecular functions of *SNB*, we performed RNA-seq experiments using 0- to 4-cm young panicles from the wild type and the *ssh1* mutant. We identified a total of 2402 differentially expressed genes (DEGs), including 1548 upregulated and 854 downregulated DEGs (fold change ≥ 2, FDR < 0.001; Supplemental Data Set 1). Further Gene Ontology (GO) and Kyoto Encyclopedia of Genes and Genomes (KEGG) analyses revealed that these DEGs were enriched in multiple biological processes, including the regulation of metabolic processes, regulation of gene expression, transcription regulator activity, plant hormone signal transduction, and phenylpropanoid biosynthesis (Figure 8A), suggesting that *SNB* is involved in a complex network regulating rice inflorescence development.

Previous studies have shown that lignin content is an important determinant of seed shattering in rice and fruit dehiscence in Arabidopsis (Mele et al., 2003; Yoon et al., 2014, 2017; Lee et al., 2018). Notably, *SH5* was previously found to negatively regulate the expression of *CINNAMYL ALCOHOL DEHYDROGENASE*, an important gene for lignin biosynthesis, by directly binding to its promoter, thus affecting seed shattering in rice (Yoon et al., 2014). Eleven genes involved in lignin biosynthesis, including *CINNAMYL ALCOHOL DEHYDROGENASE*, were upregulated in the young panicles of the *ssh1* mutant (Figure 8B).

To determine whether *SNB* suppresses lignin deposition in the AZ, we detected the AZ lignin contents of the wild type, the *ssh1* mutant, and the transgenic plants at spikelet developmental stage Sp8 using phloroglucinol staining. Lignin deposition was lower in the wild-type, GC-*SNB*, and OE-*SNB* plants than in the *ssh1* mutant and RNAi-*SNB* plants (Figures 8C to 8G). Thus, *SNB* positively regulates the expression of *SH5* and *qSH1*, suppressing lignin deposition in the AZ and thereby modulating seed shattering in rice.

### The *ssh1* Mutant Has Larger Seeds and a Higher Grain Yield Than the Wild Type

**Figure 9.**
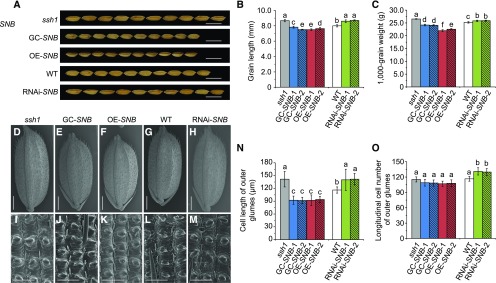
The *ssh1* Mutation Positively Regulates Seed Size and Grain Weight. **(A)** Comparison of seed size between the wild type, the *ssh1* mutant, and the GC-*SNB*, OE-*SNB*, RNAi-*SNB* transgenic plants. Bars = 1 cm. **(B)** Grain length of the wild type, the *ssh1* mutant, and the GC-*SNB*, OE-*SNB*, and RNAi-*SNB* transgenic plants. Values are means ± sd (*n* = 30 grains). **(C)** The 1000-grain weight of the wild type, the *ssh1* mutant, and the GC-*SNB*, OE-*SNB*, and RNAi-*SNB* transgenic plants. Values are means ± sd (*n* = 10 plants). **(D)** to **(H)** SEM images of grains from the wild type, the *ssh1* mutant, and the GC-*SNB*, OE-*SNB*, and RNAi-*SNB* transgenic plants. Bars = 1 mm. **(I)** to **(M)** SEM images of the lemma outer epidermis cells from the wild type, the *ssh1* mutant, and the GC-*SNB*, OE-*SNB*, and RNAi-*SNB* transgenic plants. Bars = 100 μm. **(N)** and **(O)** Comparison of the longitudinal cell lengths and cell numbers of the outer glumes in the wild type, the *ssh1* mutant, and the GC-*SNB*, OE-*SNB*, and RNAi-*SNB* transgenic plants. Values are means ± sd (*n* = 10 grains). In **(B)**, **(C)**, **(N)**, and **(O)**, different letters denote significant differences (P < 0.01) determined using Tukey’s honestly significant difference analysis (Supplemental File 2).

In addition to the decreased shattering observed in the *ssh1* mutant, these plants were also found to have increased seed lengths (+7.0%) and 1000-grain weights (+6.1%) compared with the wild-type plants (Figures 9A to 9C). To determine whether the mutated *SNB* gene positively modulated seed length, we investigated the seed size and grain weights of the transgenic plants. We found that, compared with the controls, the GC-*SNB* and OE-*SNB* lines had shorter seed lengths and lower 1000-grain weights, while downregulating the expression of *SNB* (RNAi-*SNB*) resulted in longer seeds and dramatically increased 1000-grain weights (Figures 9A to 9C). Further histological examination using SEM revealed that both the mutation and the downregulation of *SNB* significantly increased the longitudinal lengths of outer epidermis cells in the lemma. By contrast, the overexpression of *SNB* decreased the cell lengths of these tissues (Figures 9D to 9N). The numbers of outer epidermis cells in the lemma showed no significant increase in the *ssh1* mutant relative to the wild type (Figure 9O), indicating that *SNB* regulates the seed size mainly by modulating the longitudinal cell lengths. In addition, an evaluation of other yield-related traits showed that, although the *ssh1* mutant had fewer primary branches and a lower seed set ratio, *ssh1* had an increased number of secondary branches, resulting in a significant increase in overall grain yield (Supplemental Figure 11).

To investigate whether the introgression of the EMS-generated *ssh1* allele into the current cultivars would have a positive effect on grain weight, we introduced the *ssh1* allele into a nonshattering *indica* variety, 93-11, by backcrossing it with the *ssh1* mutant. We then selected 10 plants homozygous for the *SSH1* allele and 10 plants homozygous for the *ssh1* allele from the BC_1_F_2_ population and measured their seed lengths and weights. The 93-11*^ssh1^* plants had increased seed lengths (+9.5%) and 1000-grain weights (+7.7%) compared with the 93-11*^SSH1^* plants (Supplemental Figure 12), suggesting the *ssh1* allele as a possible target for efforts to enhance grain yields.

### Nucleotide Diversity and Selection Signature in *SNB*

**Figure 10.**
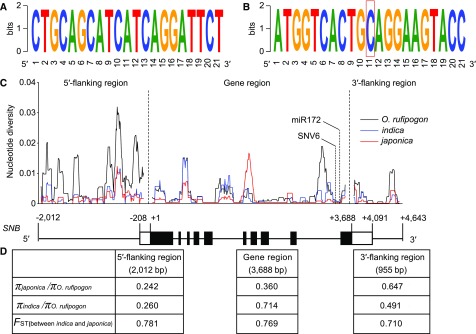
Nucleotide Diversity and *indica*-*japonica* Differentiation Analysis of *SNB* using 46 Accessions of *O. rufipogon*, 48 *indica* Varieties, and 38 *japonica* Varieties. **(A)** Conservation analysis showing that both wild and cultivated rice lines have identical sequences at the miR172 target site. **(B)** Conservation analysis of the 10-bp upstream and downstream sequences surrounding the SNP SNV6, showing that both wild and cultivated rice have a conserved C at the SNV6 site. The SNP SNV6 is boxed in red. **(C)** Sliding-window analysis of nucleotide polymorphism (*π*) in *SNB*. The values were calculated for each sliding window of 100 bp with an increment of 10 bp. Black boxes represent exons, white boxes represent UTRs, and thin black lines indicate flanking regions and introns. **(D)** The values of *π_japonica_*/*π_O. rufipogon_*, *π_indica_*/*π_O. rufipogon_*, and *F*_ST_ (between *indica* and *japonica*) were each calculated for the 5′-flanking region, gene region, and 3′-flanking region of *SNB*.

To investigate the nucleotide diversity of *SNB*/*SSH1* in wild and cultivated rice, we sequenced a 6655-bp genomic fragment covering the entire *SNB* gene (3688 bp), a 2012-bp 5′-flanking region, and a 955-bp 3′-flanking region, from 46 accessions of *O. rufipogon* (the wild ancestor of Asian cultivated rice) and 86 Asian rice cultivars (48 *indica* and 38 *japonica* cultivars collected from 16 countries). Nucleotide alignment showed that the wild and cultivated rice had identical sequences at both the miR172 target site and the SNV6 SNP identified in this study (Figures 10A and 10B). We also recovered the reads that mapped to the miR172 target site and the 21 bp flanking the SNP SNV6 from publicly available genome resequencing data of 446 accessions of the wild rice species *O. rufipogon* and 1083 cultivated *indica* and *japonica* varieties (Huang et al., 2012). The sequence alignment of these reads showed that both the cultivated and wild rice genomes had highly conserved sequences at these two sites (Supplemental Figures 13A and 13B), which was consistent with the results generated using Sanger sequencing. However, the nucleotide diversity at the 5′-flanking region of *SNB* was strongly reduced in cultivated rice compared with wild rice (Figure 10C). The percentage of nucleotide diversity at the 5′-flanking regions between *japonica* and *O. rufipogon* and between *indica* and *O. rufipogon* was 24.2 and 26.0%, respectively (Figure 10D), much lower than the average levels observed using random gene fragments across the rice genome (42% in *japonica*/*O. rufipogon* and 48% in *indica*/*O. rufipogon*, respectively; Caicedo et al., 2007), suggesting that the 5′-flanking region of *SNB* was subjected to selection during rice domestication. In addition, the fixation index (*F*_ST_), which was used to evaluate the genetic divergence between populations, was more than 0.7 at the coding, 5′-flanking, and 3′-flanking regions of *SNB* between *japonica* and *indica* (Figure 10D). We also analyzed the *F*_ST_ on chromosome 7 using the SNP data between geographically diverse cultivated rice (Huang et al., 2012). The *F*_ST_ level in *SNB*/*SSH1* was higher between the *indica* and *japonica* subspecies than it was between the temperate *japonica* and tropical *japonica* subpopulations (Supplemental Figures 13C and 13D); therefore, we propose that differences in *SNB*/*SSH1* might exist between *indica* and *japonica* subspecies.

## DISCUSSION

The AP2/Ethylene Responsive Element Binding Factor gene family contains plant-specific transcription factors with one or two conserved AP2 domain(s), which are divided into two major lineages: euAP2 and AINTEGUMENTA (Riechmann and Meyerowitz, 1998; Kim et al., 2006; Zumajo-Cardona and Pabón-Mora, 2016). The *AP2*-like genes execute a series of floral development functions, including specifying the identity of the floral organs and modulating the expression of genes related to flower development (Kim et al., 2006; Ripoll et al., 2011; Samad et al., 2017). The *AP2*-like genes also play important roles in fruit dehiscence in Arabidopsis (Ripoll et al., 2011) and seed shattering in wheat and rice (Simons et al., 2006; Zhou et al., 2012; Debernardi et al., 2017). In the present study, we found that SNB, a transcription factor belonging to the euAP2 group and closely related to maize SID1, regulates seed shattering by conferring abscission layer identity, suppressing lignin deposition in AZ, and repressing vascular bundle overgrowth in rice. In addition, the *ssh1* mutant identified in this study represents a new mutant allele of the rice heterochronic gene *SNB*, which was previously reported to regulate the transition from the spikelet meristem to the floral meristem (Lee et al., 2007; Zhu et al., 2009; Lee and An, 2012). These findings support the suggestion that the AP2 transcription factors have at least partially conserved functions in regulating the development of the AZ and inflorescences in dicots and monocots.

Previous studies have shown that the Arabidopsis *AP2* gene (*At4g36920*) negatively regulates the homeobox gene *RPL*, which controls the development of the replum, a structure associated with pod dehiscence and seed dispersal (Ripoll et al., 2011). In rice, two *RPL* orthologs, *qSH1* and *SH5*, play an important role in the development of the AZ. The downregulation or dysfunction of *qSH1* and *SH5* resulted in an aberrant abscission layer and decreased seed shattering (Konishi et al., 2006; Yoon et al., 2014). SHAT1, another AP2-like transcription factor, is known to regulate the formation of the abscission layer. The expression of *SHAT1* in the AZ was positively regulated by *SH4*, while *qSH1* functions downstream of *SHAT1* and *SH4* to maintain their expression in the AZ, thus promoting AZ differentiation (Zhou et al., 2012). Despite this, our comparison of *SNB* expression in NIL-*SH4*-*SSH1* and NIL-*sh4*-*SSH1* did not indicate that *SH4* regulates the expression of *SNB* (Supplemental Figure 14). Consistent with the fact that *qSH1* functions downstream of *SHAT1*, we found that *SNB* positively regulates *qSH1* and *SH5* expression by directly binding to their promoters, thus controlling seed shattering. The NIL-*qsh1*-*ssh1* plants, harboring both the inactive variation in the *cis*-element of *qSH1* and the *ssh1* mutation, had a complete loss of the abscission layer (Figure 7D). Based on these results, we speculated that the *AP2* genes in Arabidopsis and rice might exhibit different regulatory patterns when modulating the expression of the *RPL* genes to promote organ abscission, because of the structural differences between the replum in Arabidopsis and the pedicel in rice.

In addition to controlling the development of the flower and the dehiscence zone, Arabidopsis AP2 negatively regulates the size and number of embryonic cells, thereby affecting seed size (Jofuku et al., 2005; Ohto et al., 2005). Compared with the wild type, the Arabidopsis *ap2* mutants have larger replum epidermal cells and form an enlarged replum (Ripoll et al., 2011). Consistent with this function in Arabidopsis, the mutation of *SNB* led to longer seed lengths and higher grain weights by increasing the longitudinal cell lengths of the glumes, suggesting this gene as a possible target for efforts to enhance grain yield. Similarly, the mutation of the *SHAT1* gene, encoding an AP2 transcription factor regulating seed shattering, also increased seed lengths in rice (Zhou et al., 2012). Additionally, several genes controlling seed size, including *GS2*/*OsGRF4* and *GL4*, also regulated seed shattering in rice (Sun et al., 2016; Wu et al., 2017). Notably, we found that the *ssh1* mutation also affected plant height and panicle patterning in rice, consistent with a previous report (Wang et al., 2015). Plant hormones are known to play important roles in plant height, seed size, and the separation of the AZ (Wang and Li, 2008; Estornell et al., 2013; Zuo and Li, 2014), and our genome-wide expression profile analysis showed that *SNB* might be involved in plant hormone signal transduction. The future investigation of the involvement of *SNB* in plant hormone signal transduction could therefore further elucidate its roles in seed shattering and inflorescence development.

Favorable natural variants of *AP2*-like genes were selected during the domestication of wheat and barley. The wheat domestication gene *Q* encodes an AP2 transcription factor with a high amino acid similarity (64%) to SNB. The selection of two mutations, a G-to-A mutation in exon 8 and a C-to-T change within the miR172 target site in exon 10, resulted in the transition from the elongated spikes and hulled grain in wild wheats to the subcompact spikes and free-threshing grains in domesticated wheats (Debernardi et al., 2017). In addition, natural variations at the miR172 target site of barley *AP2* were selected during its domestication and improvement, associated with the transition from noncleistogamy and subcompact spikes to cleistogamy and dense spikes (Nair et al., 2010; Houston et al., 2013). In the present study, we found that the level of sequence polymorphism at the 5′-flanking region of *SNB* was strongly reduced in both *indica* and *japonica* varieties relative to the wild progenitors, similar to domestication signatures for maize *teosinte branched1* (Wang et al., 1999), *teosinte glume architecture1* (Wang et al., 2005), and rice *GRAIN INCOMPLETE FILLING1* (Wang et al., 2008). We therefore speculated that the natural variations in *SNB*, especially the *cis*-element variations at the 5′-flanking region, might be associated with the domestication and improvement of seed shattering and yield-related traits in rice, which is valuable information for the further exploration of the underlying evolutionary mechanisms of these traits in the process of rice domestication.

The *ssh1* allele identified in this study has a positive effect on seed shattering and seed weight but a weak negative effect on primary branch number and seed set ratio. The application of *SNB* would therefore be challenging in crop breeding programs. To circumvent any undesirable pleiotropic effects and enhance crop production, one strategy would be to apply CRISPR/Cas9 genome editing to generate diverse alleles of the target genes and provide beneficial variations for crop breeding (Scheben and Edwards, 2018). Another approach would be to apply temporally and spatially specific promoters that optimize the expression of the target genes (Vanhaeren et al., 2016). Thus, identifying favorable alleles of *SNB* and optimizing *SNB* expression could facilitate the improvement of rice seed shattering and grain yields.

## METHODS

### Plant Materials

The wild rice (*Oryza rufipogon*) introgression line YIL100, which possesses a high-shattering trait, was derived from a cross between an *indica* variety (Teqing) and an *O. rufipogon* accession (YJCWR). The nonshattering *ssh1* mutant was identified from the M2 plants of an EMS-mutagenized YIL100 population. The four NILs, NIL-*qSH1*-*SSH1*, NIL-*qSH1*-*ssh1*, NIL-*qsh1*-*SSH1*, and NIL-*qsh1*-*ssh1*, were developed in the genetic background of *indica* variety Teqing through the marker assistance selection of the progenies derived from crosses between the *ssh1* mutant and a *japonica* variety (C418) with an inactive *qSH1* allele. For the nucleotide polymorphism analysis, 46 accessions of *O. rufipogon* and 86 Asian rice (*Oryza sativa*) cultivars were used (Supplemental Data Set 2). All plants were grown in the field at Sanya (18°N, 109°E), China.

### Phenotypic Evaluation

To evaluate seed shattering, the main panicles of 10 plants were bagged and isolated prior to heading and used to investigate the average ratio of shattered filled grains to the total filled grains on the main panicle after harvesting. For BTS, a total of 50 spikelets or grains from three panicles were measured with a digital force gauge (FGP-1; SHIMPO). A total of 10 wild-type and *ssh1* plants were bagged before heading to investigate their yield-related traits. For each line, the seed lengths of 30 filled grains were measured with an electronic digital caliper. Two independent T2 lines (10 plants per line), GC-*SNB* and OE-*SNB*, and downregulation (RNAi-*SNB*) transgenic plants were used to investigate seed shattering and yield-related traits.

### MutMap Analysis

The MutMap method was used to map *SSH1*, according to the description by Abe et al. (2012) with minor modifications. An F2 population containing 277 individuals was constructed by crossing the *ssh1* mutant with the wild-type YIL100. Second, a recessive pool and a dominant pool of DNAs were constructed by mixing equal amounts of DNA from 20 nonshattering F2 individuals and 20 easily shattering F2 individuals, respectively. Finally, both the bulked DNA samples and the two parental DNA samples were subjected to whole-genome sequencing using an Illumina HiSeq2500 platform, which was performed by BerryGenomics. Approximately 5-Gb paired-end short reads were obtained from the *ssh1* mutant and the wild-type plants, and ∼10 Gb of reads was generated from the two bulked DNA pools, respectively. These short reads were aligned to the Nipponbare reference sequence (http://rice.plantbiology.msu.edu/) to enable the identification of reliable SNPs. The true SNPs from the mutagenesis were further screened, and the SNP index and the Δ(SNP-index) were calculated based on the SNP information, following the method described by Lu et al. (2014).

### Histological Analysis

To observe the AZ structure, spikelets at the anthesis stage were longitudinally sectioned by hand. The sections were covered with 0.01% (w/v) acridine orange for 10 min in the dark, rinsed three times in distilled water, and then observed with a 488-nm and a 543-nm laser line using an Olympus FV1000 laser scanning microscope, as described by Briggs and Morris (2008). To analyze lignin deposition, young panicles (0–4 cm) at spikelet developmental stage Sp8 were collected from wild-type, *ssh1* mutant, and transgenic plants and then dehydrated, embedded, spliced, rehydrated, and stained with phloroglucinol in 20% (v/v) HCl, according to the methods described by Yoon et al. (2014).

### Scanning Electron Microscopy

The bases of mature seeds and the glume surfaces were gold plated and observed using a Hitachi S-3400N SEM at 15 kV. The longitudinal cell numbers and cell lengths of the outer glumes, the vascular bundle area, and the AZ at the pedicle junctions were measured using ImageJ software.

### Generation of Constructs and Transformation

A 7206-bp genomic fragment from the wild type, harboring the entire *SNB* gene with a 2309-bp 5′-flanking region and a 1209-bp 3′-flanking region, was amplified using the primers GC-*SSH1*-F and GC-*SSH1*-R (Supplemental Data Set 3) and inserted into the binary vector pCAMBIA1300 (http://www.cambia.org) between the *Knp*I and *Xba*I sites to form the genetic complementary construct pGC-*SNB*. The construct p*Ubi*:*SNB* harbored the wild-type *SNB* open reading frame, which was amplified using the primers OE-*SSH1*-F and OE-*SSH1*-R (Supplemental Data Set 3) and cloned into the binary vector pCAMBIA1301 (http://www.cambia.org) between the *Bma*HI and *Spe*I sites under the control of the maize *Ubiquitin* promoter. The construct p*35S*:*SNB*-*GFP* contained the *SNB* coding sequence, except that the TGA terminator was fused with *GFP* at the C terminus, driven by the *35S* promoter. An inverted repeat harboring a 323-bp fragment from the wild-type *SNB* cDNA was inserted into the vector pTCK303 (Wang et al., 2004) to develop the construct pRNAi-*SNB*. All plasmid constructs were introduced into the *Agrobacterium tumefaciens* strain EHA105. The constructs pGC-*SNB*, p*Ubi*:*SNB*, and p*35S*:*SNB*-*GFP* were transferred into the *ssh1* mutant, and the construct pRNAi-*SNB* was transferred into the wild-type plants. Primers used for all cloning are listed in Supplemental Data Set 3.

### Subcellular Localization

To determine the subcellular localization of SNB, two plasmid constructs were generated: p*35S*:*OsMADS15-RFP* (Wang et al., 2010), a nuclear localization marker, and p*35S*:*SNB-GFP*. The two plasmid constructs were cotransformed into rice protoplasts as described by Bart et al. (2006). After a 16-h incubation at 28°C in the dark, GFP and RFP fluorescence were examined with 488- and 543-nm laser lines using an Olympus FV1000 laser scanning microscope.

### Transcription Activity Assay

To generate the plasmid constructs for the transcription activity assay using the Matchmarker GAL4 Two-Hybrid System 3 (Clontech), full-length coding sequences and various truncations of *SNB* were amplified, using cDNA from the wild type as a template. The PCR products were cloned into *Eco*RI and *Pst*I sites of pGBKT7 to fuse to the GAL4 BD, and the transcription factor PROSTRATE GROWTH1 (Tan et al., 2008) was fused with the GAL4 BD as the positive control. All plasmid constructs were transformed into the yeast strain AH109 to evaluate the transcription activity of SNB, following the manufacturer’s instructions.

### RNA Extraction, RACE, and RT-qPCR

Total RNAs from various tissues were extracted using the Trizol reagent (Thermo Fisher Scientific) and purified using the RNeasy Mini Kit (Qiagen), following the manufacturer’s instructions. First-strand cDNA was synthesized using SuperScript reverse transcriptase (Thermo Fisher Scientific) with an oligo(dT)_12–18_ primer. The RACE was conducted with the 3′-Full RACE kit (TaKaRa), according to the instructions. RT-qPCR was performed using a CFX96 real-time system (Bio-Rad). The rice housekeeping gene *UBIQUITIN* (LOC_Os03g13170.1) was used as an internal control to normalize the gene expression data using the relative quantification method (2^–ΔΔCT^) described by Livak and Schmittgen (2001). Each set of experiments had three biological replicates containing a pool of cDNA from five plants.

### RNA-Seq Analysis

Total RNA was isolated from the young panicles (0–4 cm) of the wild type and the *ssh1* mutant, with three biological replicates each containing five plants. Paired-end libraries were constructed and sequenced using an Illumina HiSeq2500 at BerryGenomics. The raw reads were mapped to the reference genome (Os-Nipponbare-Refrence-IRGSP-1.0, MSU7) using TopHat2 with the default parameters (Kim et al., 2013). Cuffdiff was used to calculate the fragments per kilobase of exon per million mapped reads of each gene and identify the DEGs (fold change ≥ 2, FDR < 0.001) between the *ssh1* mutant and the wild type (Trapnell et al., 2010). The functional category analysis of the DEGs was performed using agriGO and KEGG (Yi et al., 2013; Tian et al., 2017).

### Immunoblot Analysis

Leaves of rice seedlings were ground into powder in liquid nitrogen and then suspended in protein extraction buffer (62.5 mM Tris-HCl [pH 7.4], 10% [v/v] glycerol, 2% [w/v] SDS, 2 mM EDTA [pH 7.4], 1 mM PMSF, and 5% [v/v] β-mercaptoethanol). The samples were boiled, and the supernatants were resolved on a 12% (w/v) SDS-PAGE gel. The separated proteins were transferred to a nitrocellulose membrane (GE Healthcare) and detected by immunoblotting with the antibodies Anti-SNB (CUSABIO, DK70) and Anti-HSP (Beijing Protein Innovation, AbM51099-31-PU), respectively.

### mRNA in Situ Hybridization

Young panicles (0–4 cm) were collected from the wild type and the *ssh1* mutant, then fixed in 3.7% (v/v) Formalin-Acetic Acid-Alcohol solution, dehydrated, embedded in paraffin (Sigma-Aldrich), and sliced into 8-μm sections using a microtome (Leica RM2145). Three ∼300-bp fragments of *SNB*, *qSH1*, and *SH5* cDNA were amplified and used as templates to generate sense and antisense digoxigenin-labeled RNA probes, which were prepared using a DIG RNA labeling kit (Roche). The mRNA hybridization and the immunological detection of the hybridized probes were performed as described previously (Zhang et al., 2007), with minor modifications.

### ChIP-qPCR Analysis

Young panicles (0–4 cm) of the SNB-GFP transgenic lines were collected and fixed in 1% (v/v) formaldehyde under vacuum. Chromatin was isolated from the samples using sucrose gradient centrifugation and sonicated to produce DNA fragments using a Qsonica Q700 (100% amplitude, 40 cycles of pulse-on 30 s and pulse-off 30 s). A 40-μL aliquot of the sonicated chromatin was reverse cross-linked and used as the total input DNA control. Immunoprecipitation was performed with anti-GFP (Abcam) or without any antibody as described by He et al. (2013). The amounts of immunoprecipitated genomic DNA were assayed using real-time qPCR, performed on a CFX96 real-time system (Bio-Rad) with three biological replicates (10 plants per replicate). The calculation of the relative fold enrichment was performed as described by Zhang et al. (2010). The corresponding samples without any antibodies were used as negative controls. Quantification involved the normalization of each immunoprecipitation (IP) or control (no antibodies) sample Ct to the input DNA sample Ct value to obtain a ΔCt (ΔCt IP or ΔCt control), and the relative enrichment of each fragment was calculated using 2^–(ΔCt IP–ΔCt control)^. The relative enrichment of the unrelated DNA sequence from the rice *UBIQUITIN* gene (LOC_Os03g13170.1) was set to 1 and used as an internal control to normalize the relative fold enrichment of the investigated fragments.

### EMSA

Full-length *SNB* cDNA was amplified using the primers His-SSH1-F and His-SSH1-R (Supplemental Data Set 3) and cloned into pET32a between the *Bma*HI and *Eco*RI sites. His and His-SNB recombinant proteins were expressed in the *Escherichia coli* Rosetta (DE3) strain and purified using Ni Sepharose Beads (GE Healthcare), following the manufacturer’s instructions. DNA gel shift assays were performed using the LightShift Chemiluminescent EMSA Kit (Thermo Fisher Scientific). The biotin 3ʹ end-labeled DNA fragments listed in Supplemental Data Set 3 were synthesized and annealed to be used as DNA probes, while the corresponding unlabeled DNA probes were used as competitors. Each 20-μL binding reaction contained 2 μL of biotin-labeled dsDNAs, 3 μg of His-SNB protein, 2 μL of 10× binding buffer, and 1 μL of 50% (v/v) glycerol. The binding reactions were incubated for 30 min at room temperature and then resolved by electrophoresis on 6% (w/v) native polyacrylamide gels in 0.5× Tris-borate-EDTA buffer. The biotin-labeled probes were detected using chemiluminescence, according to the instructions provided by Thermo Fisher Scientific.

### Dual Luciferase Reporter Assay

To construct the effector plasmid, the full coding sequence of *SNB* was inserted into the vector pGreenII 62-SK between the *Pst*I and *Kpn*I sites. For the reporter construct, a 2011-bp upstream fragment of *qSH1* and a 1772-bp upstream fragment of *SH5* were inserted into pGreenII 0800-LUC between the *Kpn*I and *Pst*I sites to drive the *firefly luciferase* (*LUC*) gene to get the *qSH1pro*:LUC and *SH5pro*:LUC plasmids, respectively. A *CaMV 35S* promoter-driven *Renilla luciferase* gene was used as an internal control. For each assay, 8 μg of effector plasmid DNA and 8 μg of reporter plasmid DNA were cotransformed into rice protoplasts with the polyethylene glycol-mediated method (Bart et al., 2006). After incubating for 16 h at 28°C in the dark, the relative luciferase activities were measured by the Dual-Luciferase Reporter Assay System (Promega). Three biological replicates were performed for each assay.

### Phylogenetic Analysis

The SNB protein sequence was used to perform a BLASTP search for homologs in other plant species. An amino acid multiple sequence alignment (Supplemental File 1) was conducted using ClustalX (version 2.1; Larkin et al., 2007). The phylogenetic tree of the AP2 subgroup genes was constructed by MEGA6 using the neighbor-joining method with a Jukes-Cantor model, pairwise deletion for missing data, and 1000 bootstrap repetitions (Tamura et al., 2013). The resulting tree was visualized and annotated using EvolView (Zhang et al., 2012).

### Sequencing and Data Analysis

The fragments covering the coding region (3688 bp), the 5′-flanking region (2012 bp), and the 3′-flanking region (955 bp) of *SNB* were amplified using six PCR primer pairs and sequenced using the Sanger sequencing approach. The nucleic acid multiple sequence alignment was conducted using ClustalX (Larkin et al., 2007). Sequence conservation of the miR172 recognition site and the SNP SNV6 site identified in this study was assessed using WebLogo (Crooks et al., 2004). Both the average proportion of pairwise differences per base pair and the *F*_ST_ were calculated using DnaSP (version 5.1; Librado and Rozas, 2009) . In addition, a previously published data set of 1034 diverse rice accessions (550 *indica*, 409 temperate *japonica*, and 75 tropical *japonica* lines) was used to calculate the *F*_ST_ across chromosome 7 between different subspecies using VCFtools, with a 100-kb window size (Danecek et al., 2011; Huang et al., 2012).

### Primers

The primers used in this study are listed in Supplemental Data Set 3.

### Statistical Analysis

The two-tailed Student’s *t* tests used to compare data from two groups and the Tukey’s honestly significant difference analyses used to compare multiple groups were performed using SPSS version 17 (SPSS).

### Accession Numbers

Sequence data from this article can be found in the GenBank/EMBL data libraries under the following accession numbers: *SNB*/*SSH1*, LOC_Os07g13170; *SH4*/*SHA1*, LOC_Os04g57530; *qSH1*, LOC_Os01g62920; *SH5*, LOC_Os05g38120; *SHAT1*, LOC_Os04g55560; *SH-H*, LOC_Os07g10690; and *OsMADS15*, LOC_Os07g01820. The RNA-seq data derived from the wild type and the *ssh1* mutant have been deposited in the National Center for Biotechnology Information’s Gene Expression Omnibus under accession number GSE116422.

### Supplemental Data

**Supplemental Figure 1.** Genotype of the wild rice introgression line YIL100.
**Supplemental Figure 2.** Comparison of *SNB* expression in RNAi-*SNB* transgenic plants and the controls.
**Supplemental Figure 3.** Full-length cDNA of *SNB* and the deduced amino acid sequence.
**Supplemental Figure 4.** Phylogenetic tree of the *AP2* subgroup genes from rice and other plant species.
**Supplemental Figure 5.** Floral morphology in the *ssh1* mutant.
**Supplemental Figure 6.** Positions of the 3′ end of *SNB*/*ssh1* in the wild type and the *ssh1* mutant.
**Supplemental Figure 7.** Chromosome positions of seed shattering-related genes in rice.
**Supplemental Figure 8.** Overexpression of *SNB*-*GFP* fusion gene rescues the mutation phenotype of the *ssh1* mutant.
**Supplemental Figure 9.** Screening of putatively bound sites in the promoters of *qSH1* and *SH5* using an EMSA.
**Supplemental Figure 10.** Graphical genotypes of four NILs in the genetic background of the *indica* variety teqing.
**Supplemental Figure 11.** Comparison of yield-related traits in the wild type and the *ssh1* mutant.
**Supplemental Figure 12.** The *ssh1* allele increases seed lengths and weights in the *indica* variety 93-11 background.
**Supplemental Figure 13.** Consensus sequences at the miR172 target site and the SNV6 site in *SNB* and the *F*_ST_ on chromosome 7 detected using publicly available genome data from wild and cultivated rice.
**Supplemental Figure 14.** Comparison of *SSH1* expression in two NILs, NIL-*SH4*-*SSH1* and NIL-*sh4*-*SSH1*.
**Supplemental Table 1.** SNP information in the *SSH1* mapped region.
**Supplemental Data Set 1.** DEGs between the wild type and the *ssh1* mutant detected using RNA-Seq.
**Supplemental Data Set 2.** Plant materials used in this study.
**Supplemental Data Set 3.** Primers used in this study.
**Supplemental File 1.** Text file of the alignment used for the phylogenetic analysis in Supplemental Figure 4.
**Supplemental File 2.** The results of statistical analyses.

## Dive Curated Terms

The following phenotypic, genotypic, and functional terms are of significance to the work described in this paper:

MYB3 Gramene: AT1G22640
MYB3 Araport: AT1G22640

SDS Gramene: AT1G14750
SDS Araport: AT1G14750

SHA1 Gramene: AT5G63780
SHA1 Araport: AT5G63780

At4g36920 Gramene: AT4G36920
At4g36920 Araport: AT4G36920

AP2 Gramene: AT4G36920
AP2 Araport: AT4G36920
